# Draft Genome Sequence of New Leprosy Agent *Mycobacterium lepromatosis*

**DOI:** 10.1128/genomeA.00513-15

**Published:** 2015-05-21

**Authors:** Xiang Y. Han, Nipun A. Mistry, Erika J. Thompson, Hong-Li Tang, Kanhav Khanna, Li Zhang

**Affiliations:** a Department of Laboratory Medicine, The University of Texas M. D. Anderson Cancer Center, Houston, Texas, USA; b Department of Bioinformatics, The University of Texas M. D. Anderson Cancer Center, Houston, Texas, USA; c Sequencing and Microarray Facility, The University of Texas M. D. Anderson Cancer Center, Houston, Texas, USA

## Abstract

*Mycobacterium lepromatosis* is a newly discovered cause of leprosy. Here, we present a near-complete genome of *M. lepromatosis* from strain FJ924 obtained from a patient who died of leprosy. The genome contained 3,215,823 nucleotides and matched ~87% with the *Mycobacterium leprae* genome. This genome is likely the smallest of all mycobacterial genomes known to date.

## GENOME ANNOUNCEMENT

Leprosy is caused by the well-known *Mycobacterium leprae* (1) and the newly discovered *Mycobacterium lepromatosis* (2). The bacilli differed at least 9.1% and diverged ~10 million years ago from their last common ancestor (3, 4). This difference contrasts with the clonal nature of worldwide *M. leprae* strains (5–7). Several studies have shown that *M. lepromatosis* is the long-elusive second agent of leprosy (8–14). The organism has been identified so far in leprosy patients from Mexico (8–10), Canada (11), Brazil (12), Burma (12), and Singapore (13). It is likely the dominant cause of leprosy in Mexico (9). Dual infections with both *M. lepromatosis* and *M. leprae* have been described (8, 12, 13). Like *M. leprae*, *M. lepromatosis* has not been cultivated in medium so far.

The present draft genome of *M. lepromatosis* was sequenced from strain FJ924 that was purified and enriched initially from autopsy liver tissue (2, 3). Due to exhaustions of the fresh organism and extracted DNA, the sequenced DNA was extracted from scraped bacilli on a glass slide smear that had been dried, stained (Kinyoun method), and archived for 6 years. The extraction yielded ~3 ng DNA by use of the QIAamp kit (Qiagen, Valencia, CA). A whole-genome library was then constructed using the KAPA kit (Kapa Biosystems, Wilmington, MA), enriched by six PCR cycles, and sequenced on the HiSeq 2000 sequencer (Illumina, San Diego, CA).

Sixty-nine million reads with paired ends were generated. The reads were filtered to remove human sequences (14 million, ~20%) and matched to the closest *M. leprae* Br4923 genome (5) with BLAST v2.2.26 (14) for enrichment and removal of contaminant bacterial DNA. The matched reads (11 million) were assembled *de novo* (Velvet v1.2.10) (15); the contigs were aligned manually and through use of Bowtie 2 v2.1.0 (16) to the *M. leprae* genome for orders, orientations, and gap closure. A tentative draft genome resulted, which was refined through GapFiller v1-10 (BaseClear BV, Leiden, The Netherlands) (17) to capture unique sequences from all the 69 million reads. Eventually, a draft genome consisting of 3,215,823 nucleotides from 39 final contigs was obtained. From the 11.5 million mapped reads, 500-fold coverage of the genome was achieved. As a quality indicator, the genome contained all 20 genes and pseudogenes (22,814-bp) that were sequenced previously from the same strain (3). This assessment and the low number of gaps hinted that this draft genome was nearly complete.

This *M. lepromatosis* genome matched ~87% overall with the *M. leprae* genome (3,268,071 nucleotides) (4, 5). Being also 52 kb (~1.6%) smaller, it was likely the smallest of all mycobacterial genomes known to date. This genome should complement another draft *M. lepromatosis* genome (3,206,741 nucleotides of 126 contigs) reported several weeks earlier by a separate team (18). Decoding the genome of *M. lepromatosis* should be useful for the research and care for leprosy.

### Nucleotide sequence accession numbers.

This whole-genome shotgun project has been deposited at DDBJ/EMBL/GenBank under the accession no. LAWX00000000. The version described in this paper is version LAWX01000000.
